# Recombinant Dengue 2 Virus NS3 Helicase Protein Enhances Antibody and T-Cell Response of Purified Inactivated Vaccine

**DOI:** 10.1371/journal.pone.0152811

**Published:** 2016-04-01

**Authors:** Monika Simmons, Peifang Sun, Robert Putnak

**Affiliations:** 1 Viral and Rickettsial Diseases Department, Naval Medical Research Center, Silver Spring, Maryland, United States of America; 2 Division of Viral Diseases, Walter Reed Army Institute of Research, Silver Spring, Maryland, United States of America; Institut Pasteur of Shanghai, CHINA

## Abstract

Dengue virus purified inactivated vaccines (PIV) are highly immunogenic and protective over the short term, but may be poor at inducing cell-mediated immune responses and long-term protection. The dengue nonstructural protein 3 (NS3) is considered the main target for T-cell responses during viral infection. The amino (N)-terminal protease and the carboxy (C)-terminal helicase domains of DENV-2 NS3 were expressed in *E*. *coli* and analyzed for their immune-potentiating capacity. Mice were immunized with DENV-2 PIV with and without recombinant NS3 protease or NS3 helicase proteins, and NS3 proteins alone on days 0, 14 and 28. The NS3 helicase but not the NS3 protease was effective in inducing T-cell responses quantified by IFN-γ ELISPOT. In addition, markedly increased total IgG antibody titer against virus antigen was seen in mice immunized with the PIV/NS3 helicase combination in the ELISA, as well as increased neutralizing antibody titer measured by the plaque reduction neutralization test. These results indicate the potential immunogenic properties of the NS3 helicase protein and its use in a dengue vaccine formulation.

## Introduction

Dengue is a rapidly emerging mosquito-borne (*Aedes aegypti*) viral infection of humans, with an estimated 2.5 billion people at risk worldwide. It has been estimated that the dengue viruses (DENVs) cause 50–100 million clinically apparent infections and up to 50,000 deaths each year [1]. DENVs are members of the family *Flaviviridae* and are comprised of four antigenically related serotypes (serotypes 1–4). Most clinical infections result in a self-limited, acute febrile illness called dengue fever (DF), however, several hundred thousand cases of severe life-threatening dengue hemorrhagic fever (DHF) and dengue shock syndrome (DSS) also occur annually. The risk of DHF and DSS appears to be increased by the presence of antibodies from a previous dengue infection. This is hypothesized to be due to antibody-dependent enhancement (ADE) of infection by preexisting “enhancing” antibodies which form immune complexes capable of increasing viral infection in Fc receptor bearing monocytic cells and macrophages [2]. Due to the risk associated with secondary infections, a successful vaccine candidate would have to confer effective protection against all four serotypes simultaneously.

After more than 70 years of effort, a successful dengue virus (DENV) vaccine remains an elusive goal. Several groups are currently evaluating live attenuated DENV vaccine candidates in Phase 2 and Phase 3 clinical trials [3–9]. Major obstacles for the development of live virus vaccines include low seroconversion rates, prolonged immunization schedules, and sometimes, vaccine reactogenicity. As an alternative, non-replicating vaccines have been developed that could potentially shorten the dosing schedule and provide a safer preparation that can be administered to children, chronically ill or immunosuppressed individuals. The recently licensed Vero cell-derived purified inactivated vaccine (PIV) for Japanese encephalitis for example induced high-titer and long-lasting neutralizing antibody responses within two months [10]. A purified inactivated DEN-2 virus (PIV) vaccine candidate was developed, which contains the DENV capsid (C), premembrane (prM), and envelope (E) antigens, along with smaller amounts of non-structural protein 1 (NS1)[11]. This vaccine was tested in rhesus macaques where it was demonstrated to elicit virus neutralizing antibodies and protect against wild-type virus challenge three months after vaccination. A virus neutralizing antibody titer of 1:80 was estimated to be the minimum titer required for protection. In a subsequent study in rhesus macaques, a tetravalent DENV (TDENV) PIV administered on a 0, 30-day schedule, resulted in neutralizing antibody responses against all four DENV serotypes 1 month after the second dose[12].

A recent report describes the protective antibody responses of a tetravalent DENV (TDENV) PIV against all four DENV serotypes in rhesus macaques [13]. In this study, animals received 2 ug (0.5 ug per serotype) of TDENV adjuvanted with 0.1% alum on days 0 and 28. All animals had a peak neutralizing antibody titer one month after the second dose against each of the four DENV serotypes. Groups of animals were challenged with live DENV-2 or DENV-1 on days 252 (32 weeks post-dose 2) and 308 (40 weeks post-dose 2) respectively. There was no measurable viremia after DENV-2 challenge and only 0.2 mean days of viremia in the group that was challenged with DENV-1. However, most animals had detectable RNA in their serum (RNAemia) over several days after challenge indicating sterile immunity was not achieved. The authors commented that vaccine-induced cell mediated immunity (CMI) may play a critical role in reducing viral load after infection. While these results suggest that the DENV PIV vaccine elicits high-titered virus neutralizing antibodies, it might not be as effective at eliciting cell-mediated immune responses and conferring long-term protection [14]. Therefore, the addition of the DENV nonstructural protein 3 (NS3), which is a potent stimulator of cell-mediated immunity, might significantly improve the efficacy of the PIV vaccine and provide longer term protection [15].

**Fig 1:**
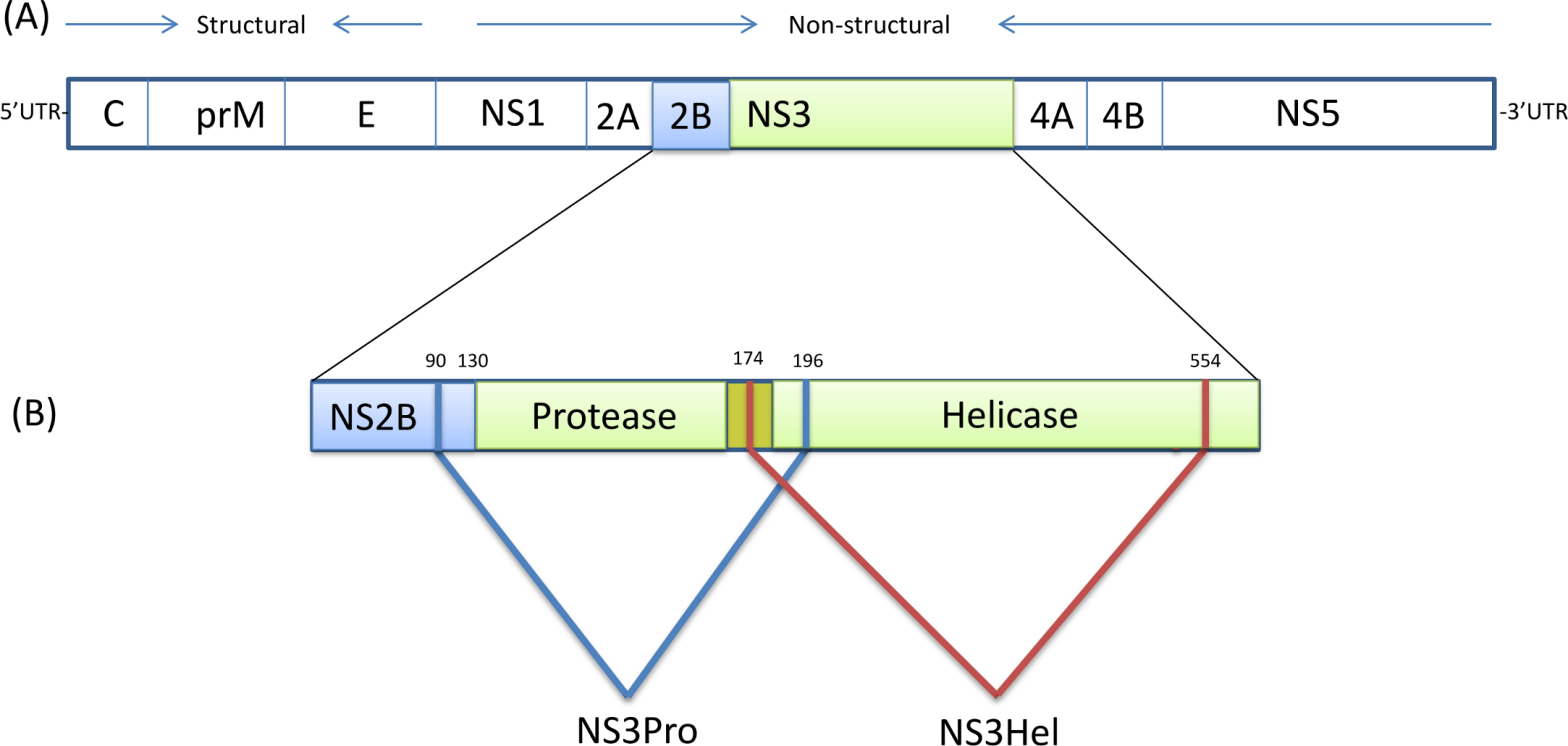
**Schematic diagram of the DENV-2 genome (A) and the regions coded by the different recombinant plasmids (B).** Dengue virus is a single-stranded, positive-sense RNA genome of about 11 kb in length. The open reading frame encodes the structural proteins capsid (C), membrane (M), envelope (E) and seven nonstructural proteins (NS1, NS2A, NS2B, NS3, NS4A, NS4B and NS5). The region linking the protease domain and helicase domain of NS3 (residues169 to 179) are shown in yellow. The last 40 residues of NS2b (90 to 130) were added to the NS3 protease (NS3Pro) fragment (1 to 196). The NS3 helicase (NS3Hel) fragment contains residues 174 to 554.

The NS3 protein is 618 amino acids (aa) in length containing serine protease and helicase domains required for DENV replication [16–19], and at least 30 T-cell epitopes, 14 (47%) of which are clustered within a 124 aa-long stretch from aa 200 to aa 324 (Fig 1). Alignment of consensus amino acid sequences from all four DENV serotypes demonstrated that this region is more conserved (78%) than NS3 as a whole (68%), leading the authors to conclude that NS3-derived peptides could be targets for reactivated, DENV cross-reactive T-cells primed by previous infection. Neither the breadth nor the magnitude of the T-cell responses was associated with the serotype of the infecting virus. Recombinant NS3 proteins from hepatitis C virus (HCV) and Bluetongue virus (BTV), which are closely related to dengue, have also been shown to generate potent T-cell responses [20–23]. Jiao et al. [21] showed that co-delivery of recombinant HCV NS3 protein and CPG oligodeoxynucleotides can induce strong immune responses against HCV NS3 and that this formulation has the ability to switch the immune response from the Th2 to the Th1 pathway. Gao and colleagues [22], using a HCV-NS3 Th1 minigene vaccine, reported enhanced CD4+ Th1 cell responses in vivo, and Wuest et al.[24], using an ovine adenovirus (OAdV) vector coding for HCV-NS3, demonstrated the induction of NS3-specific, IFN-gamma secreting T-lymphocytes after a single inoculation.

In the present study we evaluated the incorporation of recombinant subunit NS3 proteins representing protease and/or helicase as components of the DENV-2 PIV vaccine to determine if they generate better T-cell responses than the DENV PIV vaccine alone.

## Materials and Methods

### Ethics Statement

The research protocol using animals in this study was reviewed and approved by the Walter Reed Army Institute of Research/Naval Medical Research Center Institutional Animal Care and Use Committee (IACUC) in compliance with all applicable Federal regulations governing the protection of animals in research. Housing and experimental use of the animals were performed in strict accordance to all applicable Federal regulations governing the protection of animals in research. The animals were housed in static micro-isolator (filter top) cages (Lab Product, Naywood, NJ) containing Alpha-Dri paper bedding (Sheperd Specialty Papers, Kalamazoo, MI), which were changed under a laminar flow hood at least two times per week. Commercial rodent ration (Rodent Lab Chow, #5001C, Ralston-Purina, St. Louis, MO) and water from individual water bottles was provided ad lib. No more than 6 adults were housed in a single standard-sized cage. Terminal exsanguination of mice was achieved by axillary bleeds. Mice were lightly anesthetized with Ketamine/Xylazine (Ketamine (100 mg/ml) at a dose of 80 mg/kg mixed with Xylazine (20 mg/ml) at a dose of 20 mg/kg) prior to retro-orbital sinus bleeds and heavily anesthetized with (Ketamine(100 mg/ml) at a dose of 240 mg/kg mixed with Xylazine (20 mg/ml) at a dose of 60 mg/kg) for the terminal axillary bleeds.

### Virus

Cell culture supernatant harvested from Vero (ATCC, CCL-81) cells infected with DENV-2 (S16803)) (Dr. K. Eckels, Walter Reed Army Institute of Research, Silver Spring, MD) was used as virus stock to prepare antigen for the enzyme-linked immunosorbent assay (ELISA) and for the plaque reduction neutralization test (PRNT).

### Construction of Expression Plasmids

Expression plasmids containing a His_6_ tag at the C terminus were constructed as follows. Viral RNA was isolated from DENV-2 (S16803) infected Vero cell supernatant. Primers used for the NS3 protease (NS3Pro) fragment were: forward 5’-CATATGGAAGAACA-AACACTGACCATACTC-3’ and reverse 5’-GCGGCCGCATGGAGGTCCATGATG-gtcagtc-3’ containing *Nde* I and *Not I* restriction sites, respectively. These primers generated a 708 bp fragment containing the last 40 aa of NS2b as well as amino acids 1–196 of NS3 (protease domain). Primers for the NS3 helicase (NS3Hel) fragment were: forward 5’-CATATGGACAACCCAG-AGATCGAAGATGAC-3’ and reverse 5’-GCGGCCGC-GTCTGCGTAGTTGATGCCTTCAGC-3’ and generated a 1140 bp fragment containing aa 174 to aa 554 of the helicase domain. The RT-PCR reactions were performed using the SuperScript III Platinum One-Step RT-PCR kit (Invitrogen) and the products were cloned into the TOPO-TA vector (Invitrogen). The positive clones were identified, and the plasmid DNAs were digested with *Nde I* and *Not I*. The DENV-2 NS3pro and NS3hel fragments were then subcloned into the expression vector pET-30a (+) (Novagen) at the *Nde* I and *Not I* sites upstream of the C-terminal 6 x His tag. In this vector the inserts are under the control of the *lac* promoter, which is inducible with IPTG. The integrity of both constructs was verified by sequencing.

### Expression and Purification

For the expression of recombinant proteins, the *E*. *co*li strain BL21 was transformed with the expression plasmids, and cells were grown at 36°C in LB medium containing kanamycin (100 μg/ml) and 0.5% w/v glucose for 3 hours. Cultures were kept at 4°C overnight and diluted 100-fold in LB+ medium with antibiotics. Cells were further incubated at 36°C until the optical density at 600 nm reached 1.0. Cells were then centrifuged at 6000 x g and resuspended in glucose-free LB+ containing 1mM isopropylthiogalactoside (IPTG) (Sigma-Aldrich) and incubated at 30 °C for 4 hours. Cells were harvested by centrifugation and the dry pellets stored at -80 °C until use. The bacterial pellets were analyzed by electrophoresis and Western blot. For the purification, pellets were resuspended in Bugbuster extraction reagent (Novagen) containing 0.1% Benzonase and 0.01% rLysozyme and incubated at RT for 20 minutes with gentle rocking. The cellular suspension was centrifuged at 15000 x g for 15 minutes and the supernatant removed. The pellet containing inclusion bodies was resuspended in binding buffer (8 M urea, 0.5 M NaCl, 20 mMTris-HCl pH 7.9, 5 mM imidazole) and clarified by centrifugation at 15000 x g for 15 minutes at 4°C. The supernatant was loaded onto a HisPur Ni-NTA affinity column (Thermo Scientific). The column was washed with several resin bed volumes of 8 M urea, 0.5 M NaCl, 20 mMTris-HCl pH 7.9, 60 mM imidazole until the absorbance of the flow-through fraction at 280nm approached baseline. His-tagged proteins were eluted with 8 M urea, 0.5 M NaCl, 20 mMTris-HCl pH 7.9, 1 M imidazole. Peak fractions were pooled and refolded by dialysis in PBS in a stepwise manner containing urea in decreasing amounts (6 M, 4 M, 2 M, 1 M every hour and no urea overnight at 4°C). The dialyzed preparations were concentrated using centrifugal filters with a 10K membrane cut off (Amicon, Ireland). Purified proteins were analyzed by electrophoresis and Western blot. The presence of bacterial lipopolysaccharide in the purified recombinant protein preparations was determined using a Limulus Amoebocyte Lysate (LAL) colorimetric assay (Genscript). The endotoxin values for the NS3Pro and NS3Hel were 1.88 EU/mg and 1.7 EU/mg respectively.

### SDS-PAGE and Western Blot

Protein samples were mixed with SDS gel loading buffer (Quality Biologicals, Gaithersburg, MD) and heated for 10 minutes at 70°C. Samples were loaded onto a 4–12% polyacrylamide gel (NuPAGE BT, Life Technologies, Carlsbad, CA) and fractionated according to manufacturer’s instructions. Protein bands were visualized by staining with Coomassie brilliant blue R-250 (Sigma). The protein concentrations were measured using the Bradford assay (Bio-Rad). For the Western blot, protein samples were transferred from the polyacrylamide gel to a PVDF membrane using the Pierce G2 fast blotter (Thermo Scientific, Rockford, IL) for 10 minutes. The membrane was blocked with 5% non-fat dry milk in PBS/0.01% Tween 20 for 1 h at 37 °C, washed three times with PBS/0.01% Tween 20, and then incubated with DENV-2 hyperimmune mouse ascitic fluid (HMAF) at 1:100 in blocking buffer for 1 h at 37 °C. After washing, the secondary antibody was peroxidase-conjugated goat anti-human IgG (Kirkegaard & Perry, Gaithersburg, MD) diluted in blocking solution and incubated for 1 h at 37 °C. The membrane was washed again and the bands were detected by incubation with 3, 3′,5,5′-Tetramethylbenzidine (TMB) Liquid Substrate System (Kirkegaard & Perry).

### Enzyme-Linked Immunosorbent Assay (ELISA)

Virus antigen was prepared by polyethylene glycol (PEG 8000) precipitation of DENV-infected and -uninfected Vero cells. PEG adsorbed virus preparations were centrifuged at 10,000 rpm for 30 minutes and pellets resuspended in TNE. Virions and control antigen were stored at -80 °C until used. The analysis of sera from immunized mice for DENV-2 antibodies was carried out as previously described [25]. Briefly, microtiter plates were coated with DENV-2 virions in PBS at 4 °C overnight followed by blocking with 5% non-fat dry milk in PBS/0.01% Tween 20 for 1 h at 37 °C. Plates were then incubated with the test sera at twofold serial dilutions starting at 1:100 in blocking buffer for 1 h at 37 °C. The secondary antibody was peroxidase-conjugated goat anti-human IgG (Kirkegaard & Perry, Gaithersburg, MD) diluted in blocking solution and incubated for 1 h at 37 °C. The2.2’ azinodi(3ethyl benzthiazoline sulfonate (6)) (ABTS) peroxidase substrate system (Kirkegaard & Perry) was used to visualize dengue virus-specific antibody. The net optical density (OD) values were determined by subtracting the absorbance of test serum with negative control antigen from the absorbance of test serum with the DENV antigen. Endpoint dilution titers were determined by the dilution at which the OD value was at ≥ 0.10.

### Immunization of Mice

Seven groups of 20 female DBA/1J mice (Jackson Laboratory, Maine) 6–8 weeks old, were immunized by intramuscular (tibialis anterior muscle) inoculation on days 0, 14 and 28 with DENV-2 PIV (340 ng), DENV-2 PIV + NS3Pro (340 ng PIV + 75 μg NS3Pro), DENV-2 PIV + NS3Hel (340 ng PIV + 75 μg NS3Hel), DENV-2 NS3Pro (75 μg), and DENV-2 NS3Hel (75 μg) adjuvanted with 0.1% aluminum phosphate (Adju-Phos, Accurate Chemical, NY). Similarly, groups of mice received 0.1% Adju-Phos (200 ul) as a negative control, and for a positive control two doses (day 0, 14) of 10^7^ plaque forming units of live DENV-2 virus in PBS (100 ul). On days 0, 8, 22 and 35, five mice in each group were sacrificed, bled and the spleen harvested and passed through a nylon mesh to prepare single cell suspensions. Erythrocytes were lysed by brief exposure to ACK lysing buffer (155 mM NH_4_Cl, 10 mM KHCO_3,_ 190 mM EDTA). Cells were washed, viable cells counted and stored in LN_2_ until further use.

### Interferon-γ ELISPOT

For the detection of IFN-γ secreting T cells an ELISPOT assay was performed on blinded samples. Cryopreserved mouse splenocytes from day 35, inoculated with PIV, PIV-Pro, PIV-Hel, protease and helicase, as well as adjuvant (negative control) and virus (positive control) were thawed, washed, and brought to 2 × 10^6^ cells/ml in complete medium (RPMI 1640 (w/o l-Glutamine) (Mediatech, Manassas, VA), 1% penicillin, (Invitrogen, Frederick, MD), 1% l-glutamine, 1% non-essential amino acids (Mediatech), and 10% heat-inactivated fetal bovine serum (Hyclone Laboratories, Logan, Utah). Two peptide pools containing overlapping peptides derived either from the whole length of NS3 (DENV-2, New Guinea C) (BEI resources) or prM and 80% of E (DENV-2 S16803) (AnaSpec Inc, San Jose, CA) were used to stimulate T cells at a final concentration of 1μg/ml per peptide. Non-stimulated and concanavalin A (Con A 10μg/ml) stimulated cells were used as negative and positive controls, respectively.

MultiScreen MAIPSWU10 96-well plates (Merck Millipore, Cork, IRL) were coated with 100 μl/well of 10 μg/ml anti-human IFN-γ (Mabtech, Sweden) in PBS at room temperature for 3 h. The plates were washed with RPMI 1640 and blocked for 1 h. One hundred microliters of the cell suspension (2 × 10^6^ cells/ml) plus 100 μl of the peptide pool or media control were added to duplicate wells, and cultures were incubated for ∼24 h at 37°C in a 5% CO_2_ humidified atmosphere. The cell cultures were decanted, and the plates were washed six times with PBS/Tween 20 (Sigma Aldrich), followed by 2-h incubation at room temperature with 100 μl/well of the biotin-anti-IFN-γ in PBS (Mabtech). The plates were washed six times, and 100 μl of streptavidin-horse radish peroxidase (Mabtech) was added to each well for an additional 90 minutes at room temperature. For color development, the plates were washed again six times, and 100 μl/well of substrate (AEC Peroxidase Substrate Kit, 3-amino-9-ethylcarbazole) (Vectors Laboratories, Burlingame, CA) was added to each well. When reddish spots emerged, the plates were washed under tap water to end the reaction. The spots were counted using the AID ELISPOT Reader (AID Autoimmun Diagnostika, Germany). IFN-γ responses specific to the NS3, M or E peptide pools were scored as the mean number of spots in duplicate cultures after subtracting the mean background values detected in splenocytes incubated with medium only. The data were then normalized and presented as the mean of the antigen-specific spot forming units per 10^6^ cells. The SD of the mean for duplicates was <20%.

### T cell Depletions

For the CD4 or CD8 T cell depletion, the protocol provided by Miltenyi Biotec (Auburn, CA) was used. Briefly, spleen cells from Day 35, were thawed and stained with either anti-mouse CD4-FITC or CD8-FITC (BD Biosciences, NJ) for 30 minutes on ice. After staining, the cells were washed twice and then stained with anti-FITC-microbeads (Miltenyi Biotec). The cells attached to the microbeads were then separated on a magnetic column and non-attached cells were eluted. Depletion of T cell subsets was confirmed by flow cytometry. Briefly, eluted cells were co-stained with anti-mouse CD4-PerCp-Cy5.5 (L3T4) (RM4-5) and CD8a-APC (ly-2) (53–6.7) (all from BD Biosciences) on ice for 30 minutes. The stained cells were acquired on FACS Canto II with FACSDiva software and data was analyzed by comparing the percentages of CD4 or CD8 T cells before and after the process of depletion. For the measurement of IFN- γ, the eluted cells were washed three times and plated for the ELISPOT assay.

### Plaque Reduction Neutralization Assay

Plaque-reduction neutralization tests (PRNTs) were performed to measure DENV neutralizing antibodies, using a method modified from that originally described [26]. Vero cell monolayers were seeded in six-well plates (Falcon; Becton Dickinson, Lincoln Park, NJ) and incubated at 37 °C in a CO_2_ incubator. Sera from immunized mice were tested using serial twofold dilutions starting at 1:10 until an endpoint was reached. The serum dilutions were mixed with DENV-2 to obtain approximately 50 plaque forming units (PFU) per 0.2 ml, incubated at 37 °C for 30 min, then inoculated onto duplicate wells overlayed with nutrient agarose (EMEM, 2% FBS, 1% agarose). Plaques were visualized on day 4 by staining with 0.02% neutral red in Hank’s balanced salt solution. The number of plaques reported for each serum dilution was the average of the duplicate wells. The percent reduction in plaques was calculated by comparison of the results obtained with control sera from unimmunized rhesus macaques. The neutralization titer was the test serum dilution at which 50% plaque reduction occurred (PRNT _50_ titer) determined by probit analysis.

### Statistics

Primary analysis included descriptive statistics of each animals antibody and cytokine responses (e. g. geometric mean and standard deviation). All titers were log transformed to stabilize variance. The Student’s t test was used in the data analysis; however, p-values obtained were considered to represent descriptive measure of strength of evidence rather than formal statistical inference. Statistical significance was defined as P<0.05. For calculation purposes, PRNT titers <20 were given a value of 5.

## Results

### Expression and purification of recombinant NS3 proteins

**Fig 2:**
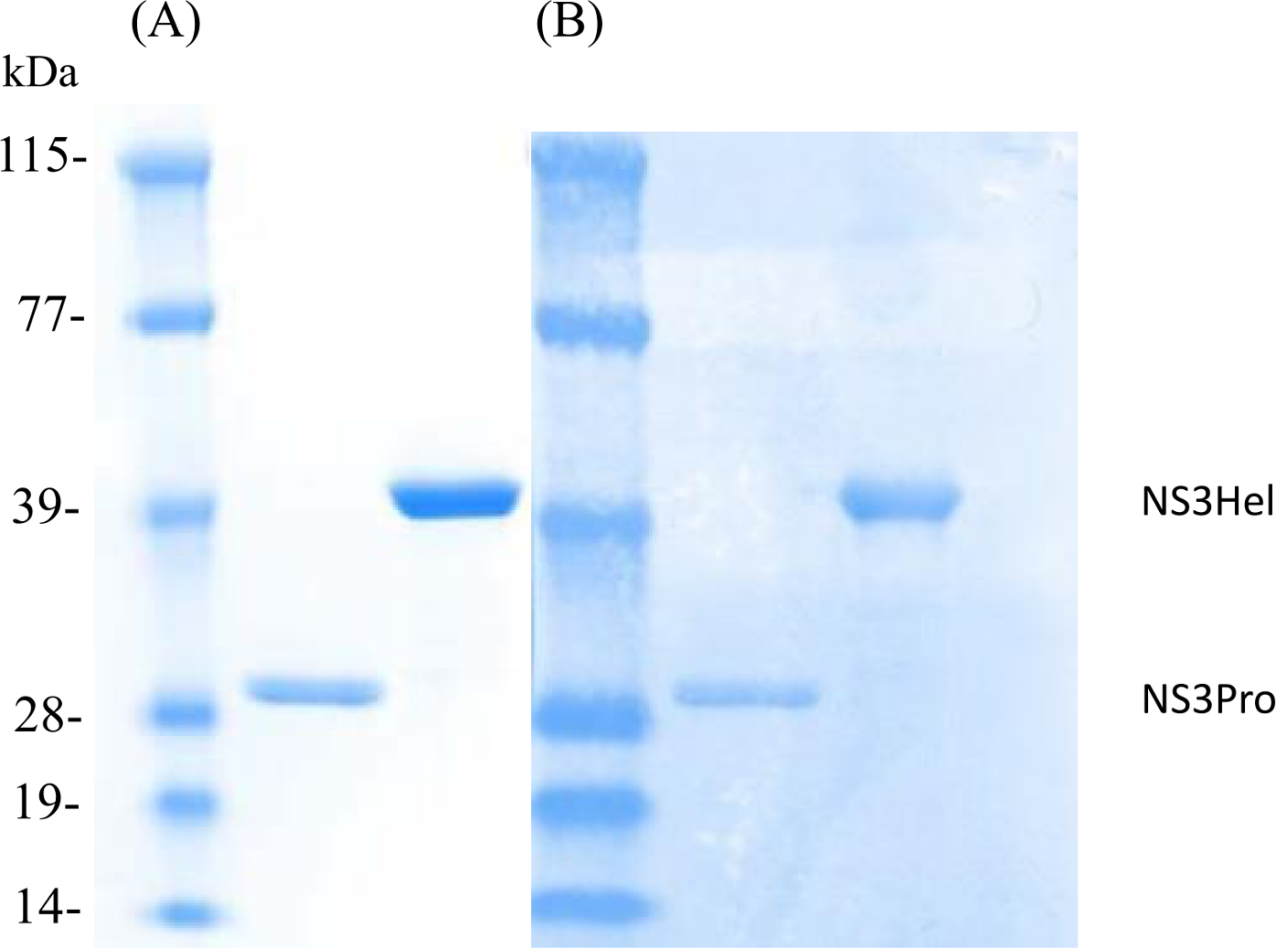
Purification and immunodetection of the recombinant NS3Pro and NS3Hel. Conditions for growth of *E*. *co*li transformed by the His-tagged expression plasmids and purification by metal affinity are described under Materials and Methods. The purified proteins were fractionated by SDS-PAGE (4–12%) and the gel was stained by Coomassie blue (A), and anti-DENV-2 HMAF was used for the Western blot (B). Lane 1, molecular size marker; lane 2, NS3Pro; lane 3, NS3Hel. Pro = protease, Hel = helicase.

The regions of the NS3 proteins encoded by plasmids are shown diagrammatically in Fig 1. The NS3Pro encodes for the C-terminal 40 amino acids of NS2B and the first 196 NS3 N-terminal amino acids, which enclose the protease domain. The NS3Hel construct encodes 380 C-terminal amino acids (from residues 174 to 554) accounting for about 87% of the helicase domain. The hexa-histidine-tagged recombinant proteins were expressed as *E*. *coli* inclusion bodie*s*, dissolved in 8 M urea and purified by Ni-NTA affinity chromatography. SDS-Page analysis (Fig 2A) and Coomassie Brilliant Blue staining showed a main band with an estimated molecular weight of ~ 30 kDa for NS3Pro and a main band of ~40 kDa for NS3Hel. Both bands were identified as DENV-2-NS3 recombinant proteins by Western blot using DENV-2 HMAF (Fig 2B).

### Antibody Responses in Mice

**Fig 3:**
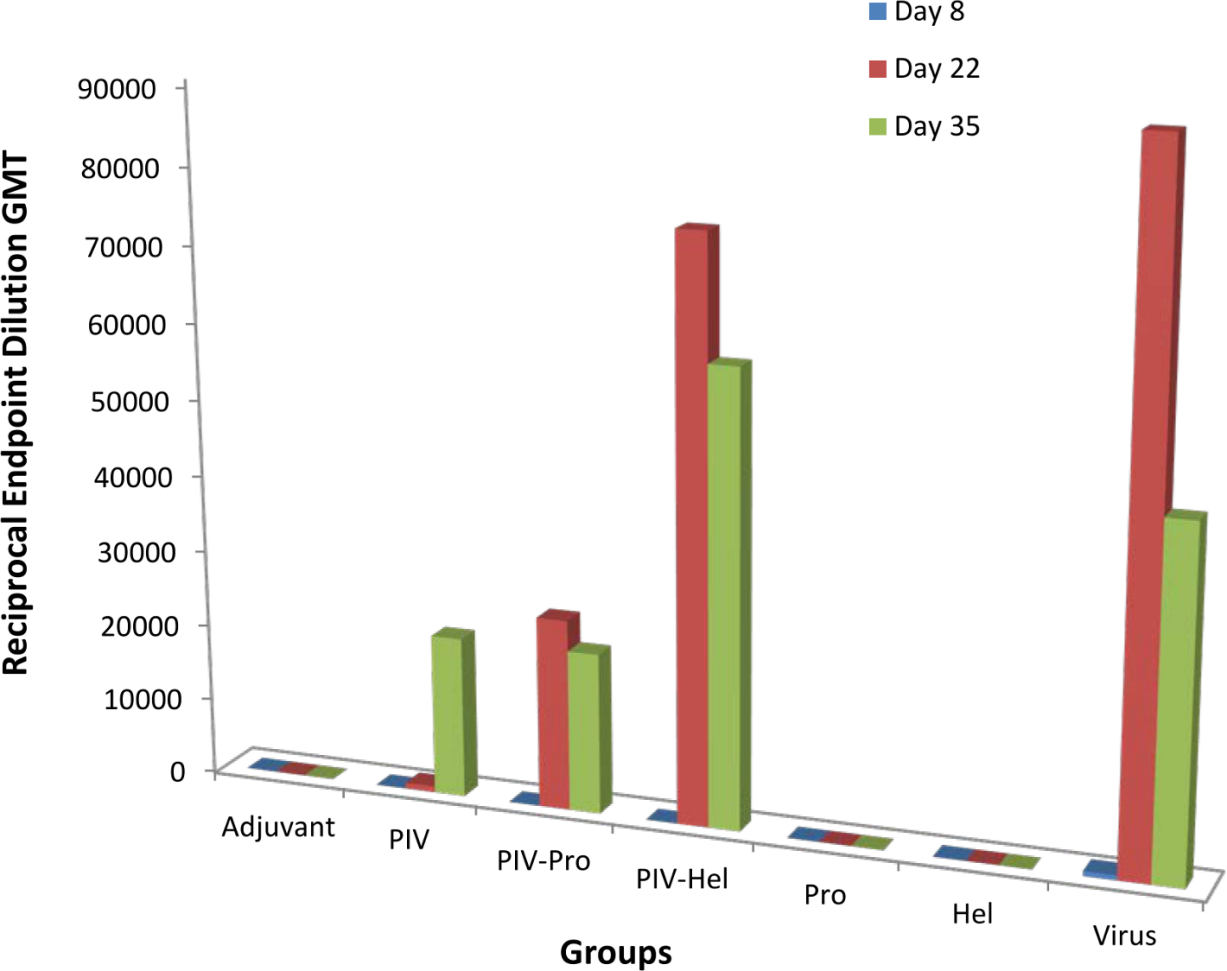
Serum IgG antibody responses. Anti-DENV-2 antibody responses in immunized mice measured by ELISA using purified virions. Bars indicate reciprocal geometric mean endpoint titers (GMT) ≥ 0.1 OD_405_. For calculation purposes titers <100 were assigned a value of 5. PIV = purified inactivated vaccine, Pro = protease, Hel = helicase.

**Fig 4:**
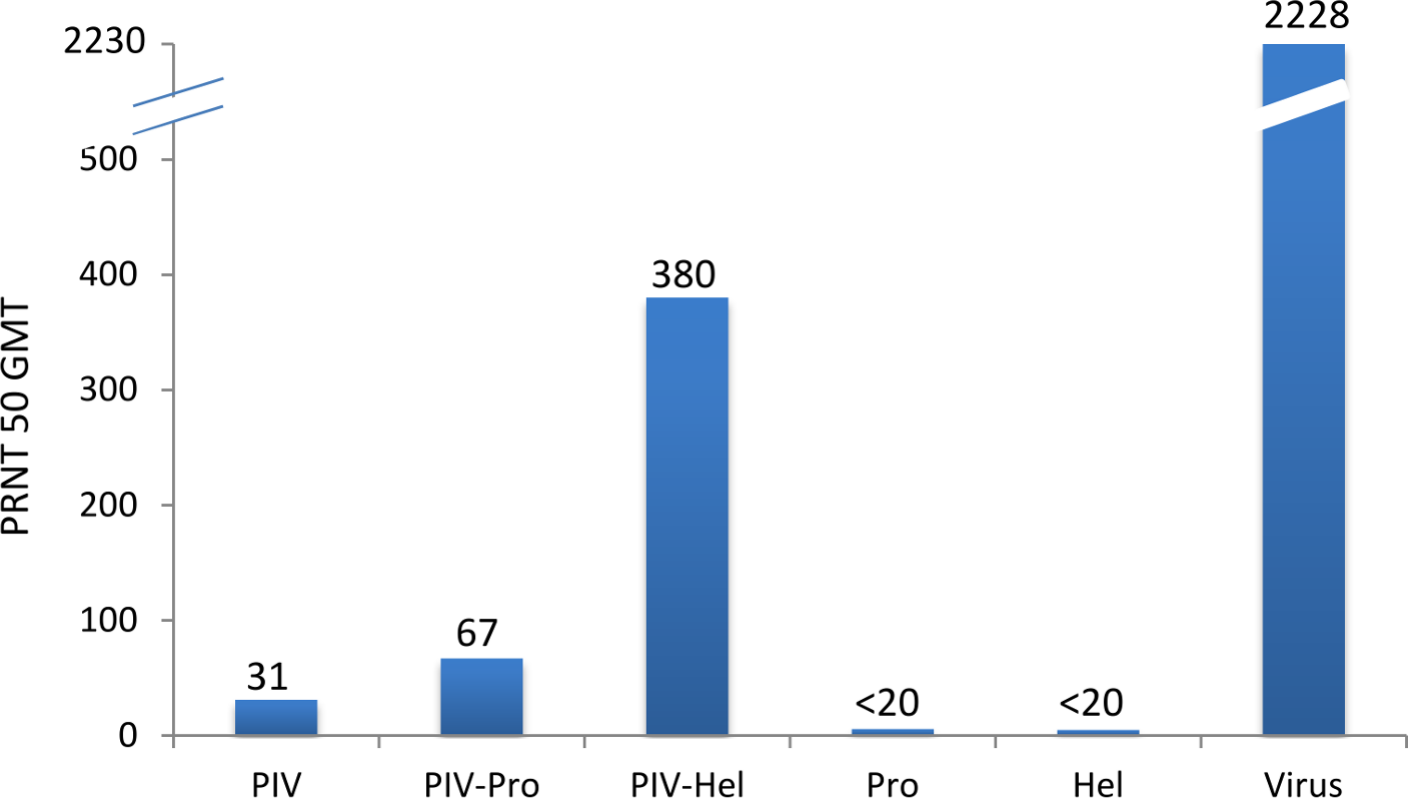
Neutralizing antibody responses. Anti-DENV-2 neutralizing antibody responses in immunized mice on Day 35 after first inoculation. Neutralizing antibody titers were determined for each animal by plaque reduction neutralization test (PRNT). The Y-axis indicates reciprocal 50% PRNT (PRNT_50_) titers (Log_10_). For calculation purposes, titers <20 were given a value of 5. PIV = purified inactivated vaccine, Pro = protease, Hel = helicase.

To determine the immunological effect of adding recombinant NS3 proteins to the DENV-2 PIV vaccine, mice were immunized on days 0, 14 and 28 with PIV alone, PIV+NS3Pro, PIV+NS3Hel, protease or helicase alone, DENV-2 (positive control) and adjuvant (negative control). Serotype specific IgG antibody titers of sera from immunized mice were measured by ELISA using DENV-2 antigen. Fig 3 shows detectable antibody on day 8 was seen only in the virus control group with a geometric mean reciprocal endpoint dilution titer of (GMT) 661. By day 22, the highest reciprocal endpoint dilution titer was seen in the virus control group (GMT = 90677), followed by the groups that received PIV-Hel (GMT = 75858), PIV-Pro (GMT = 25119) and PIV only (GMT = 794). On day 35, the GMT for the PIV only group rose to 21135, whereas all other groups indicated a slight decline in antibody titer. No antibody to DENV-2 virus was detected in the adjuvant control group, as well as the groups that received protease or helicase alone. Mice that received either protease or helicase did have IgG antibody to their respective recombinant NS3Pro or NS3Hel in the ELISA (data not shown) indicating the lack of NS3 protein in our DENV-2 antigen preparation. Neutralizing antibodies against DENV-2 were then assayed by 50% plaque reduction neutralization test (PRNT 50) on day 35 (Fig 4). A similar pattern was observed, with the virus control group showing the highest PRNT 50 GMT of 2228, followed by PIV-Hel with a GMT of 380, PIV-Pro 67 and PIV only group with a GMT of 31. There were no neutralizing antibodies greater than 1:20 detected on day 22 in any of the groups except one animal in the PIV-Hel group with a titer of 1:36. Similarly, no neutralizing antibody titers were seen in the adjuvant control group (data not shown) as well as the animals receiving protease or helicase preparations alone. Our results indicate that PIV combined with NS3Hel can generate higher total antibody as well as significantly higher neutralizing antibody titers to DENV-2 in mice than PIV administered alone (p = 0.0029).

### Production of IFN-γ Induced by NS3 Helicase

**Fig 5:**
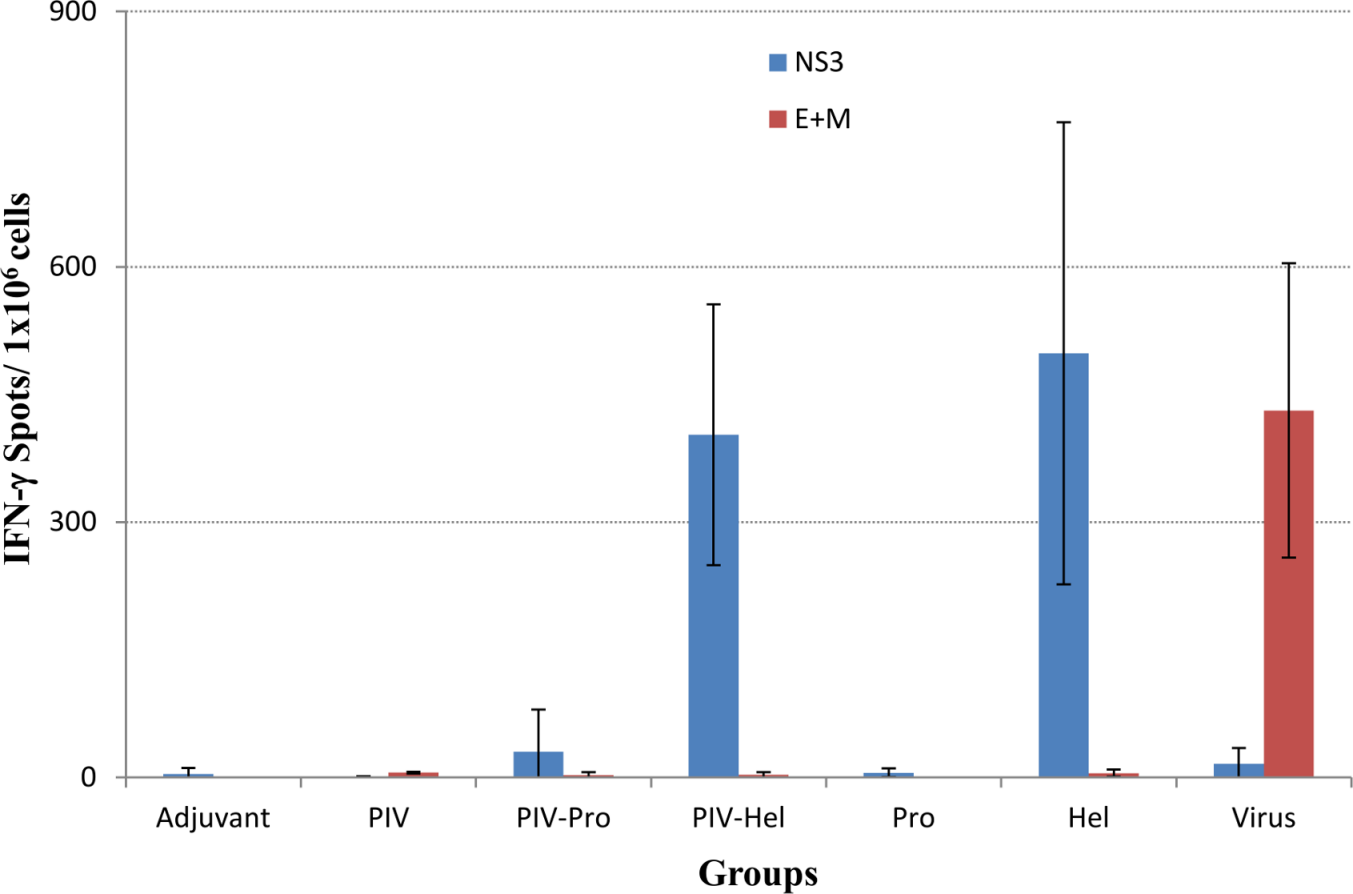
T-cell responses induced by vaccination with recombinant protein. Groups of vaccinated mice were tested for IFN-γ responses using a standard ELISPOT assay. Mouse spleen cells collected on Day 35 after first immunization, were stimulated with overlapping peptide pools spanning the entire M, E and NS3 protein. Responses are presented as number of spots per one million cells. Results indicate the mean of 3 mice with error bars equal to one standard deviation. PIV = purified inactivated vaccine, Pro = protease, Hel = helicase, E = envelope, M = membrane.

An IFN-γ ELISPOT was employed in order to investigate the T-cell response induced by the addition of recombinant NS3 proteins to the PIV vaccine (Fig 5). Mouse spleen cells were stimulated with overlapping peptides of NS3 and E protein. The highest levels of IFN-γ secreting T cells were detected in the groups that received helicase and the PIV- helicase combination (p = 0.002), whereas very few (PIV-Pro) or no spots were seen in mice that received adjuvant, PIV or protease alone. The group that received the virus control showed a low level of IFN-γ secretion when stimulated with the NS3 peptide pool but indicated a strong response with the E and M stimulating peptides. As a positive control to confirm cell viability Con A was used as a stimulating antigen (data not shown).

### CD4+ T cell Secretion of IFN-γ

**Fig 6:**
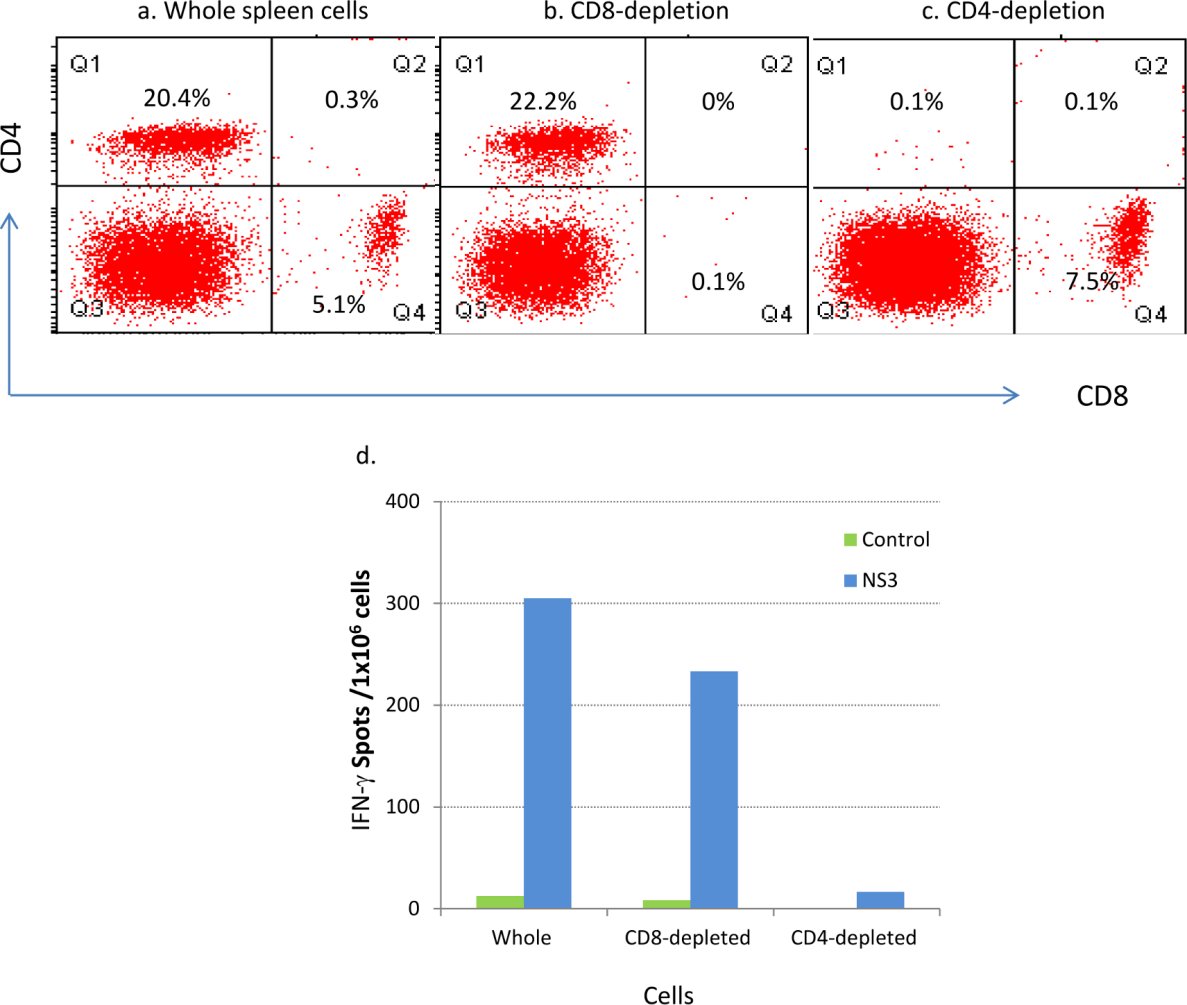
Depletion of CD4 and CD8 T cell subsets followed by IFN- γ ELISPOT. Whole spleen cells (a) or spleen cells depleted with either CD4 or CD8 subset (b, c), were stained with anti-mouse CD4 or CD8 monoclonal antibodies. The % of each subset is shown. IFN-γ response from whole or subset- depleted spleen cells was measured by ELISPOT assay (d). Results represent number of spots per million cells. Control = media without peptides.

To determine if CD4+ or CD8+ T cells are responsible for the IFN-γ secretion, we depleted CD4+ T cells or CD8+ T cells in splenocytes from PIV-Hel immunized mice and measured IFN-γ levels by ELISPOT. Whole or subset depleted cells stained with anti-CD4 or anti-CD8 monoclonal antibodies are shown in Fig 6A, 6B and 6C and the depletion process resulted in >98% of depletion of each subset. Stimulation with overlapping NS3 peptides in the ELISPOT showed a strong IFN-γ response in the CD8 depleted cells but not in the CD4 depleted cells. CD4+ T cell depleted cells had low levels of IFN-γ similar to the control (unstimulated cells) (Fig 6D). These results indicate that the observed IFN-γ secretion following co-immunization with DENV-2 NS3 helicase is due to CD4+ T cells.

## Discussion

The goal of our study was to develop a more effective purified inactivated dengue virus vaccine that would stimulate humoral as well as cell-mediated immunity. Immune responses induced by dengue virus infection include antibody responses to structural and nonstructural proteins and a variety of T cell responses. Antibodies against structural proteins prM and E have been shown to neutralize flaviviruses [27, 28] and antibodies to non-structural protein NS1 have been reported to protect mice against challenge with dengue virus [29–31]. The multifunctional protein NS3 is considered the main target for CD4+ and CD8+ T cell responses during dengue infection and maybe involved in protection [32–34]. Purified inactivated vaccines contain structural proteins C, prM, E and non-structural protein NS1, with the E protein containing most of the epitopes that elicit neutralizing antibodies [35]. How T cells contribute to the immune response in dengue disease has not been clearly defined. In a comprehensive analysis of the T cell response in Vietnamese adults, more than half recognized DENV-2 NS3 CD4+ and CD8+ epitopes [19]. This study identified 30 T-cell epitopes on the NS3 protein with 24 (80%) within the helicase domain. In our study, the immune-potentiating properties of the recombinant DENV-2 protease (NS2b-aa90 to NS3-aa196) and helicase (NS3-aa174 to aa554) as a source of T cell epitopes in combination with the DENV-2 PIV were evaluated in mice.

The recombinant protease in our study contains all 6 previously identified CD4+ and CD8+ peptide sequences of the protease domain, and the helicase contains 19 (79%) of the 24 previously identified human helicase T-cell epitopes. In addition, the recombinant helicase contains a murine CD8+ (H-2K^d^) CTL epitope at amino acids 298 to 306 (GYISTRVEM), which was previously shown to be serotype and flavivirus cross-reactive in mice [36].

The goal of an effective vaccine is to generate specific memory cells that can prevent future infections and disease. As part of the adaptive immune response, naïve T cells encounter specific antigen in the context of MHC class I (CD8+ T-cell epitopes) or MHC class II (CD4+ T-cell epitopes) molecules on the surface of antigen presenting cells (APCs). T cells proliferate and differentiate into a mixture of short-lived effector cells and long-lived memory cells. The CD4+ effector cells fall into two functional classes of helper T cells (T_H_): T_H_1 cells, whose function is to activate macrophages to eliminate intracellular pathogen; and T_H_2 cells, which activate B cells to make antibody and to promote the development memory cells. CD4+ T cells are also crucial for the generation of functional and protective antigen-specific CD8+ T cells which have the unique capability of killing infected cells and are therefore important for viral clearance.

Non-replicating virus or protein vaccines induce T-dependent responses and require adjuvants to enhance the antibody response. Aluminum salts are frequently used and are included in a majority of currently available vaccines. These adjuvants allow for the slow release of antigen, thereby extending the duration of B cell and T cell activation. The inclusion of additional epitopes on the helicase in our study may have acted as another form of immune modulator by providing additional differentiation and activation signals to monocytes and dendritic cells. This could explain the increased virus-specific IgG antibody (memory) response observed in the PIV-Hel group after the second dose, leading to a significant boost in neutralizing antibody titer after the third immunization. The addition of helicase to the PIV also resulted in activation of the cellular arm of the immune response as evidenced by the increased IFN-γ response in the PIV-Hel group. CD8+ T cell responses are elicited by vaccines that introduce antigens within the cytosol, and induction of strong CD8+ T cell responses is currently limited to infectious live-attenuated viral vaccines. For non-replicating vaccines the dose of antigen is important for the induction of memory responses. Non-replicating vaccines require sufficient antigen content in addition to adjuvantation for optimal T cell expansion during priming. The addition of a relatively high dose (75 μg) of T cell epitope(s) to the PIV may have increased the magnitude of the initial T cell expansion. For non-replicating vaccines, booster administration is then oriented towards optimization of the primary expansion phase. The persistence of memory B cells and T cells for long-term vaccine efficacy was not evaluated in this study.

The two effector arms of the immune system relevant to protection from viral disease in humans are neutralizing antibodies and cytotoxic T lymphocytes. Because T cells do not recognize intact virions, dengue virus-specific T cells would not be able to provide sterilizing immunity against viral infection. Anti-NS3 antibodies have been detected in human sera of patients with primary and secondary DENV infections [37, 38]. Studies employing a DNA vaccine based on the NS3 from Japanese encephalitis virus provided only partial protection against live virus challenge [39]. Other studies, using a DNA based vaccine encoding NS3 from tick-borne encephalitis virus, revealed absence of protection in mice, whereas vaccines encoding NS3 in addition to E2 glycoprotein indicated an increased neutralizing antibody response and IFN- γ responses induced by CD4+ T helper cell and CD8+ cytotoxic T cells [40, 41]. Although, purified recombinant NS3 proteins were able to induce strong anti-NS3 antibody responses in mice, they were not neutralizing antibodies [37, 42, 43]. This is in agreement with our results, where antibody directed against the DENV-2 protease or helicase were not able to bind or neutralize DENV-2 virus (Fig 3, Fig 4). Furthermore, Costa *et a*l. [44] reported that plasmids encoding only the NS3 protease domain did not result in protection, whereas plasmids based on the complete NS3 sequence or the helicase portion resulted in less morbidity and partial protection in mice. Similarly, the protease domain in our study did not elicit IFN- γ responses and only a minimal increase in neutralizing antibody titer was observed, whereas the helicase domain resulted in a significant increase in IFN- γ responses and neutralizing antibody titers when compared to the PIV alone. CD8 depletion analysis revealed that the IFN-γ response observed in our study is the result of CD4+ T cells. IFN-γ- producing CD4+ T cells generated by vaccination with Bacillus Calmette-Guerin (BCG) were shown to protect against *Mycobacterium tuberculosis* [45]. A protective antiviral role of CD4+ T cells were also reported for West Nile virus. In one study, CD4+ T cells were shown to help the antibody response, were important for clearance of virus from the CNS and maintaining (but not priming) the CD8+ T cell response [46]. Another study found West Nile virus-specific CD4 T cells exhibited direct antiviral cytokine secretion (IFN- γ, IL-2) and cytotoxicity sufficient for protection [47]. DENV-specific CD4+ T cells were shown to be involved in supporting B cell antibody production but also play a direct role in viral clearance through production of IFN-γ and expression of CD107a [48]. The contribution of CD4+ T cells to protect against flavivirus infection is virus-specific and may depend on the experimental system used.

In this study we report a novel finding that the addition of recombinant NS3 helicase domain to a killed DENV vaccine can increase virus-specific antibody and neutralizing antibody titers, as well as elicit cell mediated immune responses. Our results indicate that incorporating recombinant NS3 helicase protein as a component of the DENV-2 PIV may synergistically generate a more effective immune response.
